# JNK activation is responsible for mucus overproduction in smoke inhalation injury

**DOI:** 10.1186/1465-9921-11-172

**Published:** 2010-12-07

**Authors:** Won-II Choi, Olga Syrkina, Kun Young Kwon, Deborah A Quinn, Charles A Hales

**Affiliations:** 1 Department of Internal Medicine, Dongsan Hospital, Keimyung University School of Medicine, Daegu, Korea; 2 Pulmonary/Critical Care Unit, Department of Medicine, Massachusettes General Hospital and Harvard Medical School, Boston, MA, USA; 3 Shriners Burn Hospital, Boston, MA, USA; 4 Department of Pathology, Dongsan Hospital, Keimyung University School of Medicine, Daegu, Korea

## Abstract

**Background:**

Increased mucus secretion is one of the important characteristics of the response to smoke inhalation injuries. We hypothesized that gel-forming mucins may contribute to the increased mucus production in a smoke inhalation injury. We investigated the role of c-Jun N-terminal kinase (JNK) in modulating smoke-induced mucus secretion.

**Methods:**

We intubated mice and exposed them to smoke from burning cotton for 15 min. Their lungs were then isolated 4 and 24 h after inhalation injury. Three groups of mice were subjected to the smoke inhalation injury: (1) wild-type (WT) mice, (2) mice lacking JNK1 (JNK1-/- mice), and (3) WT mice administered a JNK inhibitor. The JNK inhibitor (SP-600125) was injected into the mice 1 h after injury.

**Results:**

Smoke exposure caused an increase in the production of mucus in the airway epithelium of the mice along with an increase in MUC5AC gene and protein expression, while the expression of MUC5B was not increased compared with control. We found increased MUC5AC protein expression in the airway epithelium of the WT mice groups both 4 and 24 h after smoke inhalation injury. However, overproduction of mucus and increased MUC5AC protein expression induced by smoke inhalation was suppressed in the JNK inhibitor-treated mice and the JNK1 knockout mice. Smoke exposure did not alter the expression of MUC1 and MUC4 proteins in all 3 groups compared with control.

**Conclusion:**

An increase in epithelial MUC5AC protein expression is associated with the overproduction of mucus in smoke inhalation injury, and that its expression is related on JNK1 signaling.

## Introduction

Smoke inhalation injury is a serious threat to victims of house fires, explosions, and other disasters involving fire and smoke. This type of injury alone can be lethal as shown in the Cocoanut Grove fire, in which 492 people died, most without burns [1]. In the Rhode Island nightclub fire, 95 people died (out of 350 victims and survivors of this tragedy), and 187 people were treated for smoke inhalation lung injury and burns [2]. Autopsy series from fire victims show sloughed mucosal cells and a collection of proteinaceous debris obstructing the airways [3]. There are multiple case reports in adults and children of airway obstruction due to these tracheobronchial casts [3]. The airway microenvironment is significantly altered by smoke inhalation with lung parenchymal damage occurring because of surfactant denaturation, loss of endothelial and epithelial barrier functions, and influx of inflammatory cells [4-7]. Previously we demonstrated smoke-induced mucus overproduction in a small animal model [8].

In the healthy lung, MUC1 and MUC4 are expressed on the apical surface of the respiratory epithelium. MUC5AC and MUC2 are expressed in the goblet cells of the superficial airway epithelium, whereas MUC5B is expressed in the mucosal cells of the submucosal glands [9]. Among them, MUC5AC is considered to be the predominant mucin in airway mucus [10]. Although mucus overproduction is one of the characteristics of the response to smoke inhalation airway injury, there is only limited information available on the regulation of mucus secretion in such injuries.

c-Jun N-terminal kinase (JNK) activation is required for the in vitro transcriptional up-regulation of MUC5AC in response to tobacco smoke [11]. However, the in vivo activation of JNK in the case of smoke inhalation has not yet been studied. In the present study, we used our previously established small-animal model of smoke inhalation injury [7] to determine whether the mucin genes were regulated by cotton smoke inhalation, and to test the hypothesis that smoke inhalation induces airway mucus overproduction through activation of the JNK pathway and that treatment with a JNK inhibitor could diminish airway mucus overproduction.

## Materials and methods

### Animal Preparation

This study was approved by the Massachusetts General Hospital Subcommittee on Research Animal Care and conducted in compliance with guidelines of United States Department of Agriculture Animal Welfare Act, Public Health Service Policy on Humane Care and Use of Laboratory Animals.

### Materials

The JNK inhibitor II (SP-600125) was purchased from Calbiochem (San Diego, CA). The dose was chosen on the basis of previous in vivo studies that showed 30 mg/kg inhibited JNK activity [12,13]. The mice were treated with SP600125 in dimethyl sulfoxide (Sigma Chemical, St. Louis, MO) or an equivalent amount of dimethyl sulfoxide without inhibitors 1 h after injury.

### Experimental animals

We used a modification of the established rodent model of smoke inhalation injury model as described previously [8]. Male C57BL/6, either wild-type JNK+/+ or JNK1-/- that have been backcrossed for five generations on a C57BL/6 background, weighing between 20 and 25 g were obtained from Jackson Laboratories (Bar Harbor, ME). The constructs pJNK1-/- was transfected into W9.5 embryonic stem (ES) cells. Chimeras were generated by injecting these ES cells into C57BL/6 (B6) blastocysts. Heterozygotes (+/-) were intercrossed to generate homozygous mutant mice (-/-) [14].

Animals were orally intubated with a polyethylene catheter under general anesthesia with intraperitoneal ketamine (50 mg/kg) and diazepam (5 mg/kg) while spontaneously breathing room air and then placed in the smoke chamber for 15 min. Following 15 min of smoke inhalation, animals were allowed to recover. Animals were extubated 10 min after smoke. Intubation lasted for 30 min. One hour after smoke exposure, some animals received an injection of JNK inhibitor or Dimethyl sulfoxide (DMSO) as a vehicle subcutaneously.

### Experimental design

Wild-type JNK1 -/- mice and the wild-type mice injected with the JNK inhibitor were assigned to one of 3 groups: one was the control group; mice in the second group were subjected to cotton smoke inhalation for 15 min followed by a 4-h recovery period; and mice in the third group were subjected to cotton smoke inhalation for 15 min followed by a 24-h recovery period. A JNK inhibitor dose of 30 mg/kg was selected on the basis of previous in vivo studies that showed that this dose inhibits JNK activity [8,15,16]. Four and twenty-four hours after exposure, the animals were anesthetized and killed by exsanguination. The mice in the control group were killed 4 h after extubation, and their lungs were removed en bloc. The control group mice were further divided into 3 groups: wild-type, **WC**; JNK1-/-, **JKOC**; and wild-type administered the JNK inhibitor, **JIC**. In addition, the mice subjected to 15 min of smoke inhalation followed by a 4-h recovery period were divided into 3 groups: wild-type, **WS4**; JNK1-/-, **JKOC4**; and wild type administered the JNK inhibitor, **JIS4**. The third group of mice subjected to 15 min of smoke inhalation followed by a 24-h recovery period were also divided into 3 groups: wild-type, **WS24**; JNK-1-/-, **JKOC24**; and wild type administered the JNK inhibitor, **JIS24**. Each group was assigned 7 mice, and a total of 63 mice were studied.

### Western blot analysis

For determination of MUC1, MUC4, MUC5AC, and JNK protein expression, Western blot analysis was performed with MUC1 (Abcam, Cambridge, UK), MUC 4 (Invitrogen, Carlsbad, California), MUC 5AC antibody and JNK antibodies (Santa Cruz Biotechnology, Santa Cruz, CA, and Cell Signaling Technology, Beverly, MA). Blots were developed by enhanced chemiluminescence (NEN Life Science Products, Boston, MA).

### Assessment of mucus

Paraffin-embedded samples were sectioned at 5 μm and stained with Alcian blue (AB) at pH 2.5 and periodic acid-Schiff (PAS) for the localization of acidic and neutral mucin distribution in the airway epithelium of control mice (anesthetized and intubated for 30 min while spontaneously breathing room air without smoke exposure) and in mice with smoke injury (anesthetized, intubated, and exposed to smoke for 15 min). Both wild type and JNK-1 -/- mice were allowed to recover from smoke inhalation and they were killed 4 h or 24 h after exposure. Intubation lasted 30 min in both groups. For quantitative analysis of the airway mucous secretion, all histological slides of the left lung were randomly sorted and masked before observation. The quantity of mucin production in the airway was assessed by measuring the percentage of PAS-positive cells in the airway epithelium. The numbers of PAS-positive cells were counted on longitudinal lung sections of the proximal to distal airways. Each section had 4 randomly selected regions evaluated, two segments of the proximal airway and two segments from the distal airway. A minimum of 100 sequential airway epithelial cells were counted from each region and the total number of PAS positive cells per total epithelial cells was determined for each region. These regional values were then averaged to give a final PAS score per animal. For quantitation of airway obstruction, each slide was systematically scanned using ×4 objective magnification, and for each cross-sectioned airway, a score of 0-100% was made as an estimate of the degree of luminal obstruction for each cross-sectioned airway present. A mean obstruction score was determined for each animal and then for each group [6].

### Pathology scoring

The pathological changes were compared using a modification of a previously described scoring system for pathological changes after smoke inhalation [8]. Briefly, we examined four fields (2 peripheral and 2 central) for five injurious variables on each slide. Injurious variables included 1) airway epithelial shedding, 2) airway epithelial edema, 3) increased cellularity in the airway and parenchymal tissues, 4) increased peribronchial and perivascular cuff area, and 5) alveolar atelectasis. The total lung injury score was calculated as the sum of each variable (0 for none or normal to 3 for severe).

### Lung immunohistochemistry

The paraffin sections were cut to 5 μm in thickness, mounted on silane-coated glass slides, and stored for 1 h at 60°C. The slides were deparaffinized with xylene, three times, 5 min each, and were rehydrated with graded alcohols (100, 95, 70 and 50%) for 5 min, respectively. After washing with 0.01 M phosphate buffered saline (PBS) for 5 min, the sections were digested with Proteinase K (20 μg/ml) at room temperature for 20 min, and were washed twice with distilled water for 2 min each. The endogenous peroxidase activity was blocked with 3% hydrogen peroxide (H2O2) in PBS for 5 min; the slides were rinsed twice with PBS for 5 min. Sections for positive control were treated with 3% H2O2, then washed twice with PBS. For negative controls, sections were covered with reaction buffer alone and incubated following same conditions. The sections were incubated 1.5 h with monoclonal antibody against MUC5AC (Santa Cruz Biotechnology, Santa Cruz, CA) at a concentration of 10 μg/ml. The sections were then incubated with biotinylated goat anti-mouse Ig antibody as the secondary antibody, and the antibody reactions were visualized by using diaminobenzidine as chromagen (DAKO, Carpinteria, CA). For microscopic observation, the sections were counterstained lightly with hematoxylin for one min. The quantity of MUC5AC protein production in the airway was assessed by measuring the percentage of MUC5AC positive cells in the airway epithelium. The method for evaluating the numbers of MUC5AC positive cells was same as PAS positive cell counting.

### Quantitative real-time PCR

Total RNA was isolated by the phenol and guanidine isothiocyanate method using Trizol^® ^(Invitrogen, Carlsbad, CA). Genomic DNA was removed from the extracted total RNA using the RNeasy kit (Quiagen, Austin, TX). cDNA was made with equal amounts of mRNA (2 μg), using the Superscript III reverse transcriptase (Invitrogen, Carlsbad, CA), as per manufacturer's instructions. The primer sequence for mucin genes were as follows: *MUC5AC*, 5'-ACTGTTACTATGCGATGTGTAGCCA-3' (sense) and 5'-GAGGAAACACATTGCACCGA-3' (antisense) (GenBank accession no. NM_010844); *MUC5B*, 5'-GAACGCCATATTCCCGACACT-3' (sense) and 5'-GCCCCAGGTGGAGGGACATAA-3' (antisense) (GenBank accession no. NM_028801); *MUC2*, 5'-ACGATGCCTACACCAAGGTC-3' (sense) and 5'-CCATGTTATTGGGGCATTTC-3' (antisense) (GenBank accession no. NM_023566); *MUC6*, 5'-CACACAACCAACACCAATTC-3' (sense) and 5'-TGAGAAAGGTAGGAAGTAGAGG-3' (antisense) (GenBank accession no. NM_181729); *GAPDH*, 5'-CAACTACATGGTCTACATGTTC-3' (sense) and 5'-CGCCAGTAGACTCCACGAC-3' (antisense) (GenBank accession no. NC_000072). Quantitative real-time reverse transcription polymerase chain reaction (qRT-PCR) was performed on the samples by using Applied Biosystems Assays-On-Demand primer/probe sets and TaqMan Universal PCR Mix (PE Applied Biosystems, Foster City, CA). The samples were analyzed on the Stratagene MX3000P sequence detection system under the following conditions: 94°C for 3 min, 45 cycles at 94°C for 30 s, 50°C. The fold change was determined as described in the Applied Biosystems manufacturer's instructions (4371095 Rev A, PE Applied Biosystems, Foster City, CA). Briefly, the average crossing threshold (CT) of the target genes for each group minus the average housekeeping gene (GAPDH) CT was used to determine the relative expression (ΔCT). The average ΔCT of the experimental animals (smoke inhalation) was subtracted from the average control (intubation only) ΔCT to determine the ΔΔCT. The ΔΔCT was then used in the formula 2^ΔΔCT ^to determine the fold change in mRNA expression. The upper and lower limits of fold change were determined by taking the averaged standard deviations of each experimental group through the above calculations [17,18].

### Immunofluorescence

Paraffin-embedded lung tissue samples were de-waxed in xylene twice for 5 min each time, rehydrated in an ethanol series (100-70%) for 3 min each followed by rehydration in phosphate-buffered saline (PBS) for 30 min. The rack is transferred into 200 ml of pre-warmed (94°C-96°C) Dako (DAKO, Carpinteria, CA) target retrieval solution. Following antigen retrieval, the sections were washed three times with PBS, blocked in 4% skimmed milk for 1 hr, and then stained using the kit mentioned below according to the manufacturer's recommendations but with the following modifications. Sections were incubated with the primary antibody pJNK (1 : 400, Cell Signaling Technology, Beverly, MA) at 4°C overnight and secondary antibody, Alexa488-cojugated goat anti-mouse IgG_1 _(1:2000. Invitrogen, Carlsbad, CA) for 60 minutes prior to viewing with a Nikon Eclipse E600 microscope using an NCF Fluor 40 objective lens. Visualization of the nuclei was by 4',6-diamidine-2'-phenylindole, dihydrochloride (DAPI) staining.

### Statistical analysis

Analyses were performed using SPSS (Version 13.0 software). For comparison between groups, analysis of variance(ANOVA) followed by multiple comparisons by Scheffé's test with Bonferroni post hoc analysis. Significance was set at P < 0.05. All values were expressed as means ± SE.

## Results

### Pathologic score and airway obstruction

Fifteen minutes smoke inhalation caused an increase in pathologic score in wild type mice either 4 h or 24 h recovery compared with control. The pathological scores 4 h and 24 h after smoke inhalation was significantly decreased by use of the JNK inhibitor or JNK -/-. Although the score was decreased with 24 h after recovery compared with 4 h in wild type mice, the results did not reach to statistical significant (Table 1). Mucous plugging was assessed periodic acid-Schiff (PAS) staining. The average percentage of airway obstruction with mucous plugging was decreased in JNK inhibitor treatment and JNK -/- mice. Although three was a trend to less obstruction in JNK -/- mice than JNK inhibitor, the results did not reach to statistical significant (Table 1).

**Table 1:** Pathologic score, airway obstruction, PAS and MUC 5AC positive cells in the airway epithelium.

	Intubation only			Smoke 15 min and 4 h recovery			Smoke 15 min and 24 h recovery		
**Group**	**Wild type** **(WC)**	**JNK inhibitor** **(JIC)**	**JNK -/-** **(JKOC)**	**Wild type** **(WS4)**	**JNK inhibitor** **(JIS4)**	**JNK -/-** **(JKOS4)**	**Wild type** **(WS24)**	**JNK inhibitor** **(JIS24)**	**JNK -/-** **(JKOS24)**

Pathologic score	0.5 ± 0.1	0.4 ± 0.1	0.4 ± 0.2	7.8 ± 1.6*	2.1 ± 0.4	1.1 ± 0.3	6.4 ± 1.2*	1.8 ± 0.4	0.9 ± 0.2
Airway obstruction (%)	9.4 ± 2.1	8.1 ± 1.5	9.1 ± 1.3	36.8 ± 9.1*	15.1 ± 3.4	12.1 ± 4.3	28.4 ± 5.7*	12.6 ± 4.4	11.0 ± 3.7
PAS positive cells (%)	0.4 ± 0.2	0.3 ± 0.2	0.3 ± 0.1	25.8 ± 7.8*	3.1 ± 1.4	1.9 ± 1.2	18.8 ± 3.7*	2.4 ± 1.6	1.1 ± 0.4

MUC5AC positive cells (%)	0.3 ± 0.1	0.3 ± 0.1	0.2 ± 0.1	23.0 ± 7.3*	2.8 ± 1.6	2.2 ± 0.9	17.8 ± 3.1*	3.4 ± 1.3	1.7 ± 0.9

Values are means ± SE.

* P < 0.05 versus control, JNK inhibitor, and JNK -/-.

### Smoke-induced mucus production in the airway of mice through JNK activation

**Figure 1:**
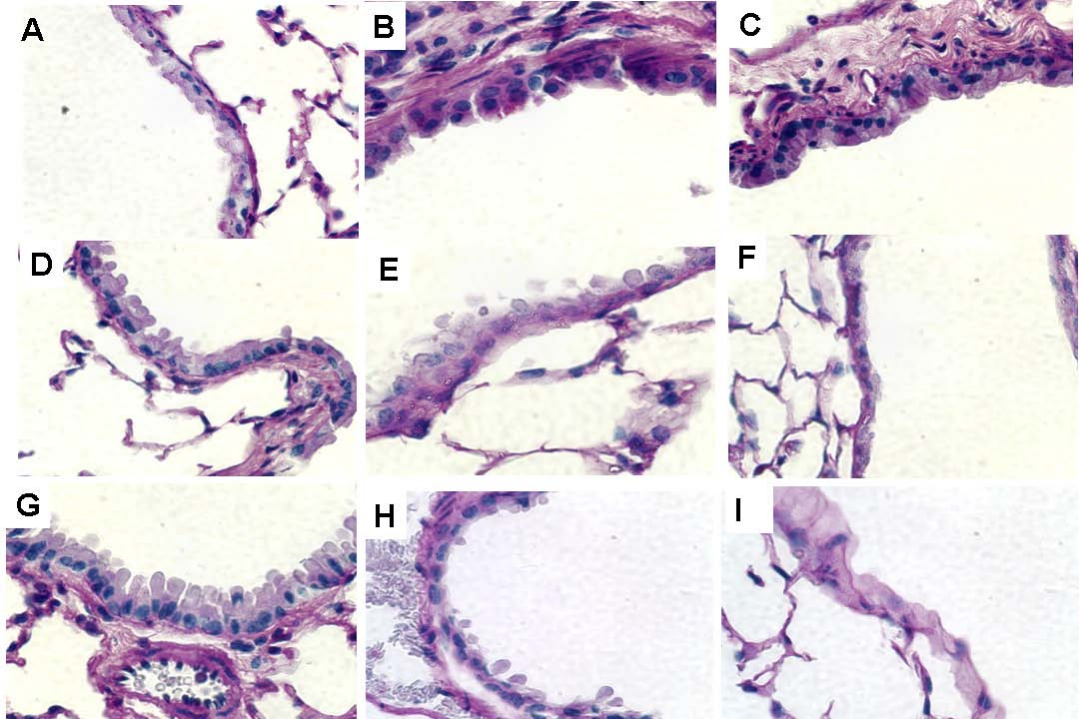
**Representative images of the airway wall stained with periodic acid-Schiff to quantify the mucin-containing goblet cells**. Histologic sections were accessed at 4 and 24 h after smoke inhalation injury. (Magnification, 400×). There was an increase in the amount of PAS-stained cells (purple-magenta color) in the small airway epithelium in the wild type mice. However, there was only minimal or no PAS staining in the mice of the control group, JNK -/- group, or JNK inhibitor treated group. A, WC; B, WS4; C, WS24; D, JKOC; E, JKOS4; F, JKOS24; G, JIC; H, JIS4; I, JIS24.

Since smoke inhalation during fires is associated with mucus hypersecretion, we evaluated mucin secretion in the airway of mice by using the PAS stain. The PAS stain is mainly used for staining structures containing a high proportion of carbohydrate macromolecules (glycogen, glycoprotein, and proteoglycans), typically found in mucus. Four and twenty-four hours after smoke inhalation, the wild-type mice clearly showed increased PAS stained cells in their airways (Figure 1). We observed minimum or no PAS staining in the mice in the control group, JNK1 KO group, and JNK inhibitor group. Semi-quantitative scale values for the percentage of PAS-positive cells were significantly increased in the WS4 and WS24 mice compared with the WC, JIC, and JKOC mice (Table 1).

### Mucin gene and protein expression

**Figure 2:**
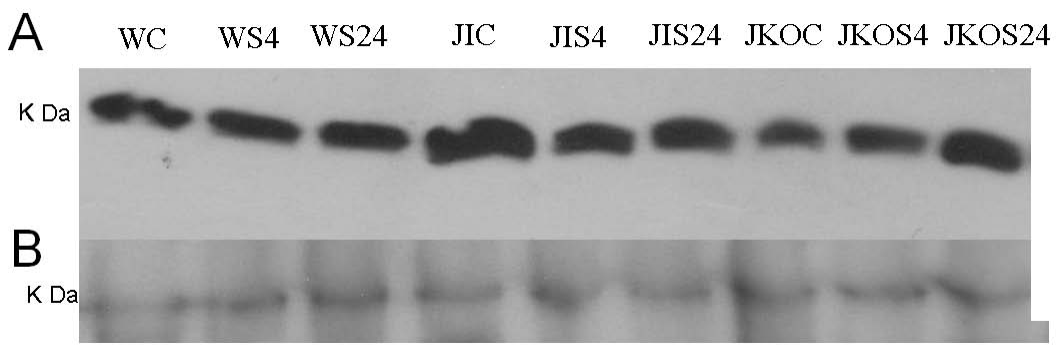
**Immunoblot of the airway and lung tissues of the mice subjected to smoke inhalation**. No difference in MUC1 (A) and MUC4 (B) (membrane-bound mucins) protein expression was observed among the 3 groups.

**Figure 3:**
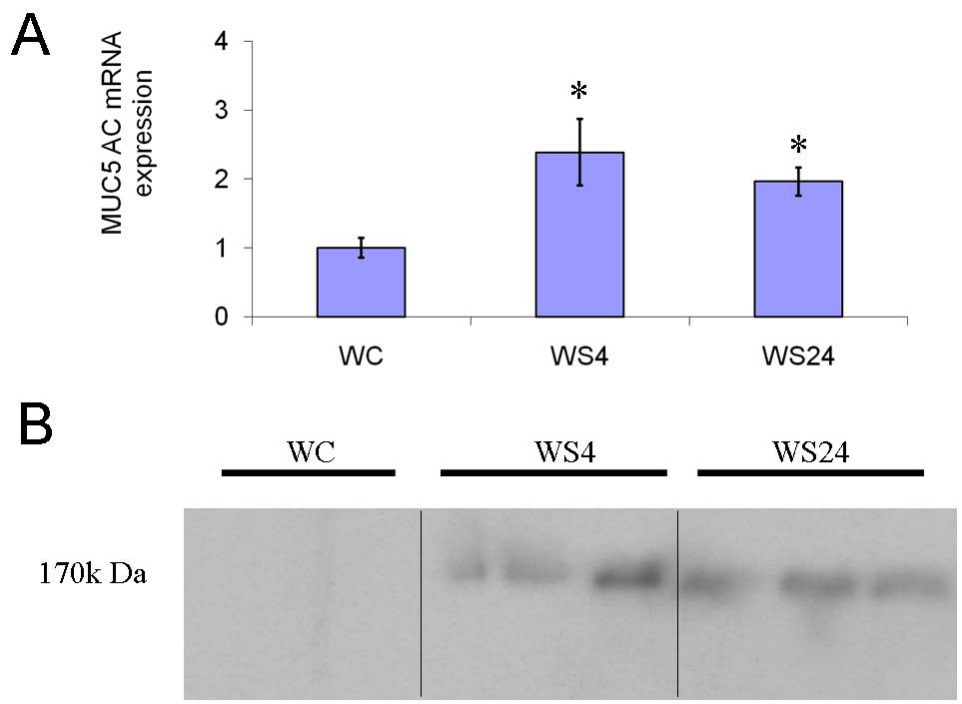
**MUC5AC RNA and protein expression**. MUC 5AC mRNA expression (A) was significantly increased in the smoke inhalation mice groups compared to the control groups. * *P *< 0.01 versus Control. Mucin protein, 170 kDa MUC5AC, expression was increased at 4 (WS4) and 24 h (WS24) after smoke inhalation injury compared with control (WC) in wild type mice (B).

**Figure 4:**
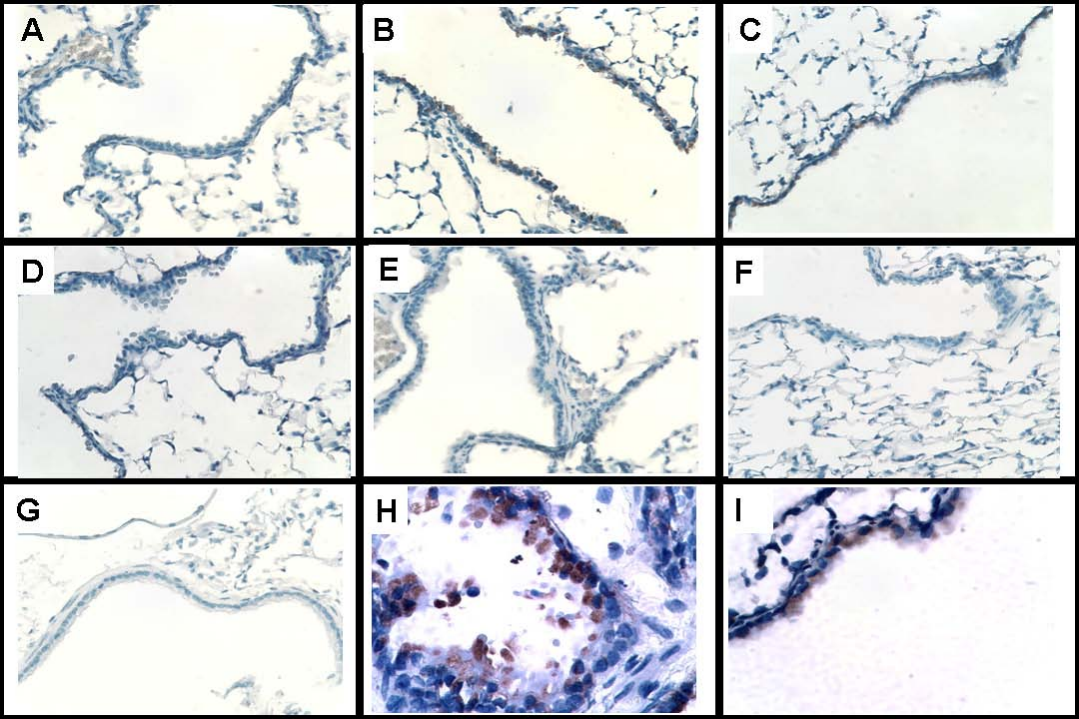
**Immunohistochemistry of the MUC 5AC protein**. Wild-type smoke inhalation mice showed increased MUC5AC protein expression in their airway epithelium 4 and 24 h after injury, whereas the JNK inhibitor and JNK -/- mice groups did not (MUC5AC protein staining: A-G, 100×; H- and I, 400×). A, WC; B, WS4; C, WS24; D, JKOS4; E, JKOS24; F, JIS4; G, JIS24; H, WS4 400×; I, WS24 400×.

MUC1 and MUC4 are important membrane-bound mucins. These mucins generate the sol layer of mucus. In the present smoke inhalation mouse model, we observed no difference in MUC1 and MUC4 protein expression between mice in the control and smoke inhalation groups (Figure 2). Gel-forming mucin genes such as MUC2, MUC5AC, MUC5B, and MUC6 were evaluated by quantitative PCR. Only MUC5AC gene expression, which was also evaluated by immunoblotting (Figure 3) and immunohistochemistry (Figure 4), was found to be increased in the wild-type mice subjected to smoke inhalation. Semi-quantitative scale values for the percentage of MUC5AC-positive cells were significantly increased in the WS4 and WS24 mice compared with the WC, JIC, and JKOC mice (Table 1).

### Smoke-induced activation of JNK

**Figure 5:**
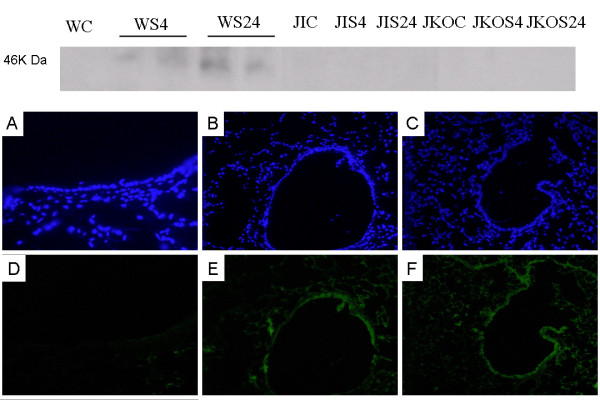
**Smoke-induced JNK activation**. Western blotting performed with an antibody that recognizes the phosphorylated form of JNK. Mice were exposed to cotton smoke for 15 min, which was followed by a recovery period of 4 and 24 h. JNK inhibitor (SP-600125) treated and JNK -/- mice did not show pJNK protein expression after smoke inhalation. Immunofluorescence (IF) showing JNK activation (D-F) in response to smoke. Green, phosphorylated JNK; blue, nuclei (4'-6-diamidino-2-phenylindole (DAPI). (Magnification, 400×). Wild-type control group mice did not show expression of *pJNK*. pJNK activation was observed predominantly in the small airway epithelium of the mice subjected to smoke inhalation at 4 and 24 h after recovery. A, WC DAPI; B, WS4 DAPI; C, WS24 DAPI; D, WC JNK IF; E, WS4 JNK IF; F, WS24 JNK IF.

Immunoblotting data suggested that *p*JNK was activated in the mice 4 and 24 h after smoke exposure (Figure 5). Immunofluorescence imaging further contributed to these results by showing that smoke induced the phosphorylation of JNK, especially in the small airway epithelium. Smoke-induced phosphorylation of JNK suggested that this kinase might participate in the induction of MUC5AC gene expression in the lung cells. To investigate this possibility, we manipulated JNK activity and assessed the effects of this treatment on the responsiveness of MUC5AC to smoke. JNK -/- or mice injected with the JNK inhibitor SP600125 attenuated both MUC5AC protein expression and JNK activity (Figure 5).

## Discussion

Airway mucus production is observed in burn trauma victims [19] and also in a combined burn and smoke inhalation injury model [6], but the mechanism by which smoke damages the airway still remains unclear. In our mouse model of smoke inhalation injury, we found that smoke inhalation induced the mucus overproduction was associated with an increase in epithelial MUC5AC protein expression, and this was dependent on the activation of the JNK pathway.

Four and twenty-four hours after exposure to smoke from burning cotton, we observed that MUC5AC mRNA levels were elevated in the mouse lungs, and MUC5AC protein was expressed predominantly in the surface cells of the mouse airway. This elevated expression was abrogated by JNK1 mutation and the JNK inhibitor, indicating the dependence of MUC5AC expression on JNK activity. JNK activation was prominent in the airway epithelial cells (Figure 5). Although the JNK inhibitor was introduced 1 h after smoke inhalation injury, we still observed a decrease in mucus production. These results suggested that the JNK pathway can be a potential target for regulating mucus overproduction in smoke inhalation injury.

In the present study, MUC5AC protein expression was increased within 4 hour after 15 min smoke inhalation. The expression was sustained after 24 hour recovery. Similar to the present study, MUC5AC can be induced within 24 hour of inflammatory or bacterial stimulation. Intratracheal instillation of IL-13 elicited huge amount of induction of MUC5AC mRNA within 24 hour in wild-type mouse lung [20]. Up-regulation of MUC5AC mucin transcription was induced by 7 hour of Streptococcus pneumoniae incubation [21]. Twelve hour of human neutrophil peptide-1 or lipopolysaccharide incubation caused an increase in MUC5AC mRNA levels [22]. However, MUC5AC can be up-regulated different time course in relation to different stimulation. In murine asthma model, airway MUC5AC gene was over-expressed after 24 hour sensitization of ovalbumin [23].

In the present mouse model of smoke inhalation, MUC5AC was the predominant gel-forming mucin gene that was expressed. We observed no differences in MUC5B, MUC2, or MUC6 mRNA expression between mice in the control and the smoke injury groups (data not shown). The membrane-associated mucins, MUC1 and MUC4, were found to be highly expressed in both the control and smoke inhalation group mice. MUC5AC gene expression was found to be increased 4 h after smoke exposure, and it remained elevated throughout the 24-h recovery period. This suggested that in the case of smoke inhalation exposure, even for short periods of time, mucus overproduction may persist for more than 24 h after initial exposure. Hence, we concluded that MUC5AC can be a potential target for reducing mucus overproduction after smoke inhalation injuries.

## Conclusions

In this study, we showed that MUC5AC protein overexpression in response to cotton smoke inhalation is tightly regulated via the JNK signaling pathways. These findings suggested that smoke inhalation can cause the overall up-regulation of MUC5AC production by JNK activation in the bronchial mucosal cells. These findings can contribute to the development of new therapeutic strategies to treat smoke inhalation injuries.

## Abbreviations

JNK: c-Jun N-terminal kinase; DMSO: Dimethyl sulfoxide; WT: wild-type; AB: Alcian blue; PAS: periodic acid-Schiff; QRT-PCR: Quantitative real-time reverse transcription polymerase chain reaction; CT: crossing threshold; GAPDH: glyceraldehyde-3-phosphate dehydrogenase; PBS: phosphate buffered saline; DAPI: 4',6-diamidine-2'-phenylindole, dihydrochloride; ANOVA: Analysis of variance.

## Competing interests

The authors declare that they have no competing interests.

## Authors' contributions

WIC was responsible for carrying out the experiments, for data analysis, and for drafted this manuscript; KYK was responsible for the analysis and design for the histologic study; OS oversaw the animal experiments, instructed WIC in his implementation; DAQ and CAH are experts in sepsis experiment and assisted in the experimental design and the data analysis and interpretation. All authors contributed to the drafting and revisions of the manuscript.
